# Implementation report on pioneering federated data access for the German National Emergency Department Data Registry

**DOI:** 10.1038/s41746-025-01481-w

**Published:** 2025-02-11

**Authors:** Jonas Bienzeisler, Alexander Kombeiz, Saskia Ehrentreich, Ronny Otto, Wiebke Schirrmeister, Marco Pegoraro, Dominik Brammen, Behrus Puladi, Rainer Röhrig, Raphael W Majeed

**Affiliations:** 1 Institute of Medical Informatics, Medical Faculty of the RWTH Aachen University, Aachen, Germany; 2 Department of Trauma Surgery, Otto von Guericke University, Magdeburg, Germany; 3 Chair of Process and Data Science (PADS), Department of Computer Science, RWTH Aachen University, Aachen, Germany; 4 Department of Oral and Maxillofacial Surgery, University Hospital RWTH Aachen, Aachen, Germany; 5 Department of Internal Medicine, Universities of Giessen and Marburg Lung Center (UGMLC), German Center for Lung Research (DZL), Giessen, Germany

**Keywords:** Health care, Health services, Public health, Medical research, Epidemiology

## Abstract

Continuous access to electronic health records will fuel the digital transformation of medicine. For data-sharing initiatives, the challenge lies in ensuring data access aligns with the interests of data holders. Federated data access authorization, where data remains controlled locally, may offer a solution to balance these interests. This paper reports on a digital health implementation of the federated data access authorization system used in the German National Emergency Department Data Registry. Using data from 2017 to 2024, we analyzed the system’s effectiveness in managing data access in a nationwide research network of 58 emergency departments. Facilitating access to more than 7.9 million records, 75% of data access queries were authorized within 15 days. The system also supports periodic queries, enabling recurring real-time access. Query volumes grew from 15 to over 23,000 by 2024, with completion rates of 86%. The system may thus serve as a blueprint for data-sharing initiatives worldwide.

## Introduction

A continuous stream of data is essential for the digital transformation of healthcare, but data collection efforts are useless if the data cannot be efficiently accessed^1–3^. The volume of clinical data stored in health information systems is constantly growing, but the benefits of holding all this data will only be realized through continuous, real-time data sharing, especially within initiatives like the European Health Data Space, which aims to create a unified system for healthcare data sharing in Europe^4^. In a learning healthcare system, technologies like digital twins or artificial intelligence rely on seamless access to electronic health records (EHRs), the patient-centered digital records of medical treatment^5^.

Providing universal access to EHRs for secondary purposes can prove challenging, as stakeholders such as physicians and hospitals may prefer to maintain control over their data^6–8^. The sharing of EHRs introduces underestimated complexities relating to data ownership, privacy, and consent^9,10^, termed the privacy-exploitation barrier^4^. Thus, access to big data for secondary purposes is increasingly expected to be facilitated by federated approaches within local healthcare settings, rather than centralized data collections^11^. Following this concept, research networks make data from nodes (local data storages, e.g., a hospital lab orders database) findable and accessible, thereby enhancing the use of health data as provisioned by the World Health Organization^12^.

The success of global data-sharing initiatives hinges on the development of processes that enable safe, efficient, and reliable data access authorization while respecting data ownership and stakeholder interests. Federated data access authorization could be a scalable solution facilitating purpose-limited data access. In this paper, we report on the digital health implementation of a nationwide federated data access authorization system for the continuous sharing of EHR data from emergency departments (following guidelines and checklists for reporting on digital health implementations^13^).

The system is used by the German National Emergency Department Data Registry (ED registry)^14^. It is the largest distributed research network in the world that uses informatics for integrating biology and the bedside data warehousing platform (*i2b2*)^15^, with 77 connected ED nodes. Emergency departments (EDs) in Germany offer both pre-hospital and in-hospital acute care services to the public and are integral to private and public hospitals. Comparable to most Western healthcare systems, they function as key hubs for urgent ambulatory and life-saving in-hospital treatment. As in other federal systems, there is a variation in healthcare regulation across the federal states of Germany.

The ED registry is based on a distributed and federated research infrastructure (AKTIN infrastructure), initiated and orchestrated by the Alliance for Information and Communication Technology in Intensive Care and Emergency Medicine, abbreviated as AKTIN in German. The infrastructure is operated within the Network University Medicine in Germany and gives access to EHRs from German EDs^14^. Records are sourced via an HL7 Clinical Document Architecture (CDA) interface from the emergency department documentation system. These CDA documents are delivered to a local AKTIN data warehouse (DWH)^16^ via a RESTful HL7 FHIR binary endpoint in accordance with the FHIR standard specification and then stored.

Each EHR corresponds to the routine documentation of an emergency department encounter episode. The stored documentation adheres to the standardized emergency department medical record^17^ of the German Interdisciplinary Association for Intensive Care and Emergency Medicine, which is developed independently of the AKTIN infrastructure in collaboration with HL7 Germany.

**Fig. 1:**
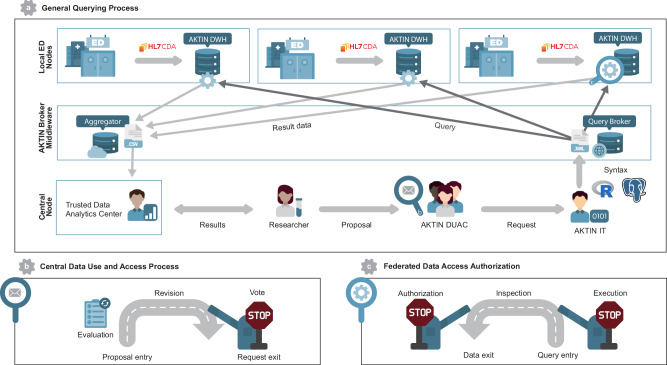
AKTIN infrastructure processes^18^. **a** Electronic health record data are captured in the participating emergency department nodes within an AKTIN data warehouse. After approval from the Data Use and Access Committee (DUAC), the AKTIN IT group translates proposals into R or SQL syntax. From the syntax, a data query is created which is communicated to the nodes using the AKTIN Broker via xml data structure including additional descriptive- and provenance metadata. The local emergency department (ED) node must authorize data access for each query. **b** The DUAC evaluates proposals and formulates a vote translated into a technical request. **c** A federated data access authorization process is implemented within the AKTIN DWH software. The AKTIN IT group receives technical requests and then uses the AKTIN Broker middleware to send queries to AKTIN DWHs in the ED nodes. The ED nodes may execute the query, inspect the results in CSV format, and authorize data access. The R logo is used under the terms of the Creative Commons Attribution-ShareAlike 4.0 International license (available from www.r-project.org/logo/). Postgres, PostgreSQL, and the Slonik Logo® are trademarks or registered trademarks of the PostgreSQL Community Association of Canada, and are used with their permission (available from www.postgresql.org/about/policies/trademarks).

The AKTIN infrastructure utilizes the open-source i2b2 data warehousing technology to empower participating EDs to integrate and manage the records exported via the HL7 CDA interface in an AKTIN DWH. The AKTIN infrastructure (Fig. 1) is composed of individual ED nodes, each representing a distinct emergency department unit within the participating hospitals operating an AKTIN DWH. These may include multiple, geographically separate EDs operating as independent organizational units in larger hospitals. While the emergency department medical record serves as a standardized ontology in all AKTIN DWHs, the selection of variables documented varies across ED nodes.

Within the framework of the AKTIN infrastructure, it is standard practice to forego the collection of informed consent for data sharing. This is possible because EHRs are stored independently within each ED, adhering to general medical confidentiality obligations. The local nodes maintain full control of their own data^18^.

Deidentified data can be queried for explicitly defined purposes following an established use and access protocol of the ED registry. A Data Use and Access Committee (DUAC) reviews data requests from scientists and enforces ethical and scientific standards^18^. The stored data can be accessed through the AKTIN Broker—a middleware service operated by the AKTIN-IT Group for querying the data stored in the AKTIN DWHs. After federated data access authorization, the queried data are collected and analyzed as a service or under supervision in a trusted research environment—the AKTIN Trusted Data Analytics Center (AKTIN TDAC).

Common ontologies facilitate data access by providing a structured framework for interoperability^19^. Universal and ongoing access, however, introduces governmental challenges if the purposes, receiver, and content of data requests (queries) vary^8,20,21^. In the AKTIN infrastructure, ED nodes must balance the interests of stakeholders when authorizing data access. The participating hospitals operate within a competitive framework. They optimize efficiency and quality in patient care to maintain a competitive advantage and reduce costs^22^. A competitive setting ensures a cautious approach towards data sharing^23^. Furthermore, hospitals may have to navigate personal interests, such as the desire of personnel to utilize data for internal research.

The decentralization of data governance introduces additional complexity. Each of the 16 German states has its own medical and general data processing regulations combined with the European General Data Protection Regulation (GDPR) provisions. While physicians operate within the scope of these laws, their responsibilities—particularly regarding the non-disclosure of medical information—are defined by the German criminal code. Data may be shared if opening clauses are in place or data is anonymized. Data protection officers are typically the process owners responsible for ensuring compliant data-sharing and the determination of applicable regulations.

Because hospitals, data protection officers, and physicians may bear liabilities, they must be equipped to make informed decisions regarding EHR sharing for secondary purposes. Research networks thus require complex frameworks for data access authorization. In the United States, PCORnet® facilitates data sharing under HIPAA^24^, while the German Medical Informatics Initiative employs a two-tiered strategy where central and local committees review proposals for data access^25^. Technical solutions, like DataShield^26,27^, MedCo^28^, and SHRINE^29^, allow the automatic querying of data. However, they lack mechanisms for a federated review process. Projects like the Norwegian PraksisNet^30^, and PopMedNet^31^, which were utilized by PCORnet® before transitioning to PCORnet® Front Door, aim to integrate data access frameworks with local control mechanisms. The efficiency of these systems in facilitating data access has neither been evaluated nor explicitly published. Here, we aim to address the research question of whether a federated data access system can effectively enable the nationwide secondary use of emergency department EHRs.

## Results

Our first objective was to design and implement a technical system and the processes required to enable secure and safe access to EHRs originating from a research network of multiple ED nodes in the AKTIN infrastructure. We then verified the operational efficiency and reliability of the technical system as our second objective. To do so, we evaluated key performance indicators (KPIs) in a retrospective cohort study based on system log data from operations between 2017 and 2024 within the AKTIN infrastructure.

In the AKTIN infrastructure, Data requests (Supplementary Fig. 1) commence with a research proposal submitted to the DUAC for review. Upon receiving authorization, the AKTIN IT group converts the proposal into a technical request. The AKTIN IT group publishes the technical request as individual queries to the nodes via the AKTIN Broker. The AKTIN DWH instances in the ED nodes poll for new queries for federated data access authorization (Fig. 1c). After authorization, which can be automatized for repeated periodic queries, the AKTIN Broker collects the results. The AKTIN TDAC retrieves the collated results. Analyses are then conducted as a service or within the trusted research environment of the AKTIN TDAC.

### Technical system

Based on our first objective, we implemented a technical system for federated data access authorization (Fig. 1c) within the AKTIN DWH Manager. The web application features a dedicated view for general data request management and for each individual query. Users can review data requests sent out as queries within the data request management view and interact with the central AKTIN Broker.

Query requests are published on the AKTIN Broker, encompassing the requisite query syntax (SQL or R Syntax), essential metadata, execution date, and a cover letter. The query is polled and visualized within an AKTIN DWH node. Users can authorize data access. Finally, the AKTIN DWH synchronizes results with the AKTIN Broker and multiple results are aggregated at the AKTIN Broker. The process is logged and managed within the backend of the AKTIN DWH and communicated to the AKTIN Broker, which logs the process status of each query for provenance. Communication between each node in the network and the AKTIN Broker is secured by using individual API Keys.

The AKTIN Broker application utilizes the original i2b2 web client for query formulation and stores queries for on-demand retrieval by AKTIN DWH nodes. Queries and, if approved, results are efficiently relayed back to the server and can then be rendered on the web frontend of the AKTIN Broker. The Broker is content-agnostic, accommodating various query languages and formats alongside their responses. Different terminologies and logics can be defined in the query through customizable XML transformations.

**Fig. 2:**
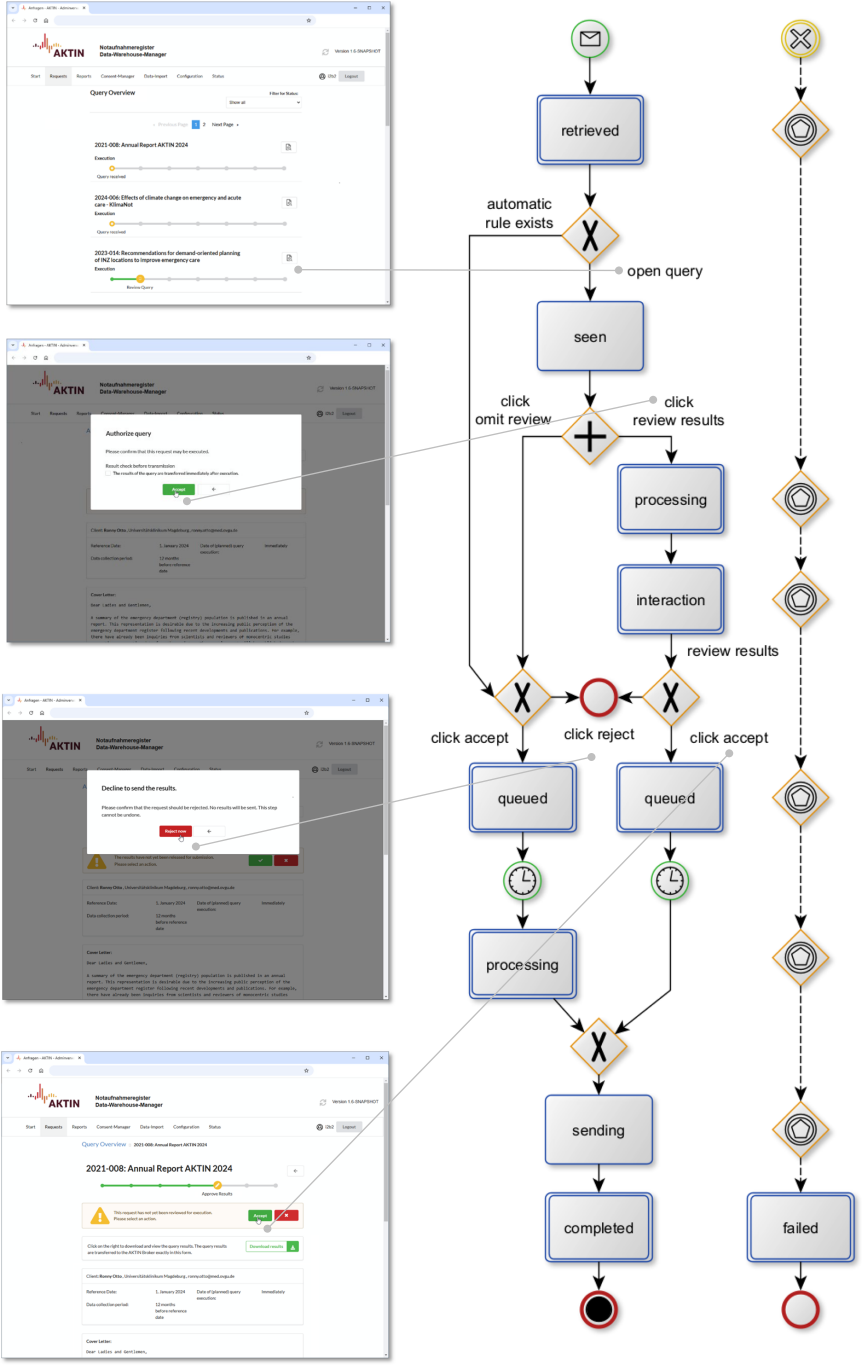
Formal workflow description and translated screenshots of local data access authorization of a query in an AKTIN data warehouse manager within a local node of the AKTIN infrastructure. Errors in the federated data access authorization process (left) result in failed queries (right). Note: this diagram employs a modified version of BPMN, adapted to clarify specific workflow elements unique to the AKTIN infrastructure. This adaptation may not strictly adhere to formal BPMN guidelines.

Within each AKTIN DWH node (Fig. 2), the request transitions through various states, offering the flexibility to approve or reject at multiple junctures. A polled request (retrieved) is displayed in the user interface. A request can then be viewed by a user (seen) and either be rejected or accepted. The request is queued until execution time (processing). Execution errors change the state to fail. After successful processing, results can be exported in CSV format. Users can also choose to review requests. The results are processed first, and an additional user interaction for result dissemination (interaction) is required. Users may also configure periodic requests to auto-execute them—a process inter alia used for pandemic surveillance^32^. After initial data access authorization, subsequent queries with identical syntax are automatically executed.

**Fig. 3:**
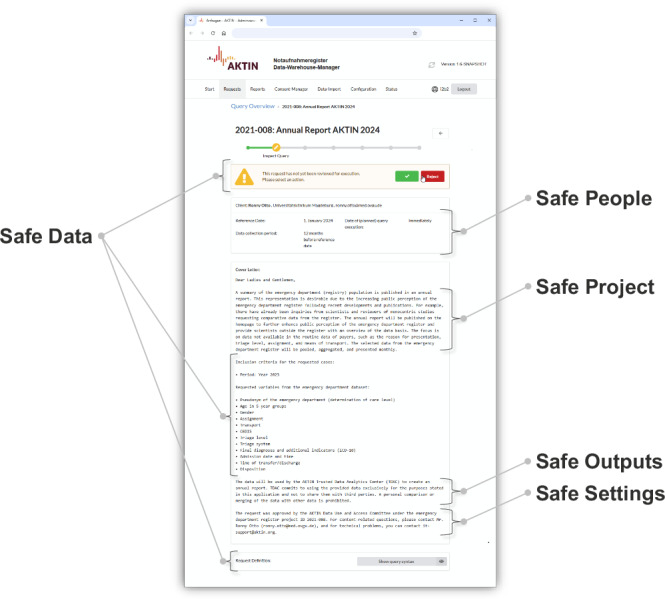
Translated visualization of a query within the dedicated data request view in the AKTIN DWH manager. Participating emergency department nodes of the AKTIN infrastructure review the query and individually decide if they want to authorize data access.

In the AKTIN DWH Manager, after user authentication, polled queries are displayed in a filterable list within the request management view. Each query triggers an email notification and is presented in a dedicated view (Fig. 3) with an ID, comprehensive meta-information, a human-readable cover letter describing purposes, and the definition of the query syntax. Meta-information consists of the requester’s details (name, affiliation, email), a reference date for the data collection period, and the planned execution. The cover letter’s content is not technically standardized but rather segmented to enhance readability. It begins with the rationale for the request, followed by a detailed account of the research questions and relevant outcomes to evaluate safe data. Inclusion criteria for the data and the requested variables are then enumerated. This is followed by a description of the data processors and the general purpose of data processing to evaluate safe settings. Standardized text elements then outline principles of the ethical use of data and general privacy commitments of the ED registry that ensure safe outputs. The described request can be approved or declined in a pop-up window and then be reviewed again. If the technical request defines a repeated query, initial approval includes a provision for automatic result transmission without subsequent authorization or rejection of future individual queries.

### System evaluation

**Fig. 4:**
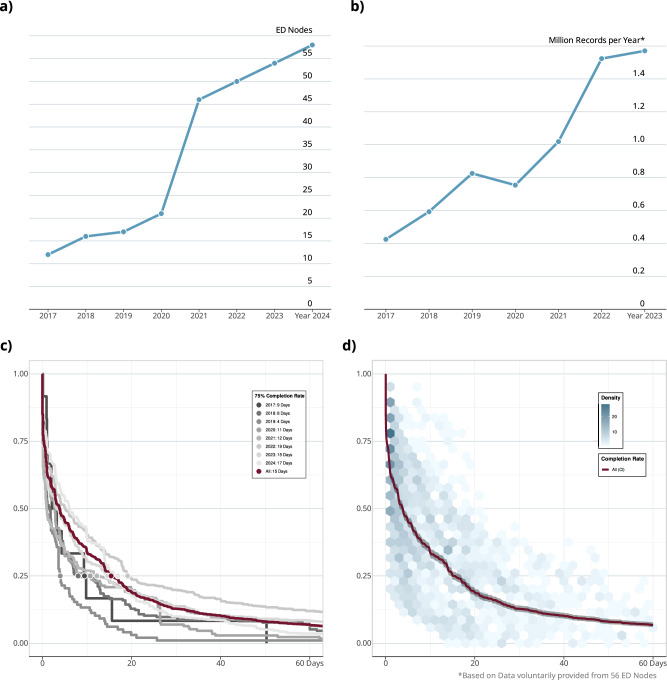
Growth of the AKTIN infrastructure and analysis of KPIs. **a** Active ED nodes connected throughout the years illustrate the infrastructure growth since 2017, which (**b**) enables access to 1.6 Million Electronic Health Records in 2023 and 7.9 Million Electronic Health Records in total. Bottom: rate of unanswered queries. Kaplan–Meier survival curves show the proportion of uncompleted individual queries over days after retrieval by the AKTIN DWH. **c** The behavior of nodes varied slightly across the years; ED nodes replied more swiftly in the first years of the infrastructure. **d** The behavior differs across nodes. The hexagonal density distribution of different Kaplan–Meier curves illustrates the concentration of query completions for all active nodes.

For our second objective, we evaluated the technical system according to KPIs. Within the time of operation, at least 7.9 million EHR datasets were integrated within the AKTIN infrastructure (Supplementary Table 2). The number of active nodes connected to the AKTIN Broker grew from 12 in 2017 to 58 in 2024 (Fig. 4). Two thousand nine hundred sixty requests, comprising 76,267 individual queries, were distributed to the nodes. This included 4053 individual queries and 72,214 periodic queries. We saw a yearly increase in query volumes, from 15 in 2017 to 23,340 queries in 2024. In 2023, each node received a median of 19 individual queries and 386 periodic queries per year.

The completion rate was 79.8% (*n* = 3234) for individual queries, 87.1% (*n* = 62,888) for periodic queries, and overall 86.7% (*n* = 66,122). Failures were infrequent, with individual queries experiencing a slightly higher failure rate of 2.8% (*n* = 114) compared to 1.0% (*n* = 722) for periodic queries. This amounted to a total failure rate of 1.1% (*n* = 836). The number of queries that were opened for review but were not interacted with was recorded at 0.1% (*n* = 3) for individual requests, and even less so for periodic queries at 0% (*n* = 16), culminating in an overall rate of 0.0% (*n* = 19). Retrieved but unanswered queries accounted for 14.2% (*n* = 574) of individual and 10.6% (*n* = 7657) of periodic queries, resulting in an overall rate of 10.8% (*n* = 8231). Rejected queries constituted 3.0% (*n* = 121) of individual queries and 1.0% (*n* = 732) for periodic queries. We observed a total rejection rate of 1.1% (*n* = 853).

Completion times varied significantly (*P* < 0.001, paired *t*-test, Supplementary Table 3) between individual and periodic queries. Individual queries took a mean of 17 days (SD: 38) to complete, compared to 9 days (SD: 23) for periodic queries. The median completion time for individual queries was 4 days (range: 0–368 days), and immediate for periodic queries. Because the automatic authorization of periodic queries can be granted to future and past queries, we observed a range from 0 day to 585 days. The time until rejection for individual queries was longer, with a median of 38 days (SD: 89). The processing time exhibited some variability, with individual queries taking a median of 21 s (IQR: 5–85) and periodic queries taking a median of around 12 s (IQR: 5–29 s).

The different nodes showed variations in response patterns to queries (Fig. 4). The response patterns were roughly similar throughout the years. Across all participating ED nodes, 75% of responses came within 15 days of delivery to the node. While most ED nodes responded to queries relatively quickly, some EDs had slower response rates. This is reflected in a prolonged tail and affects the overall mean time until completion.

There were no instances of accidental approvals or rejections. On occasion, individual inquiries were made regarding the authorization process or technical issues. None were made about the content of the queries. The system encountered downtime only once due to a certificate issue. In 2019, the AKTIN Broker server was relocated to a different university without complication.

Our results cover data from EDs across Germany, accounting for at least 1 million EHRs from some 10 million ED cases billed in approximately 1500 emergency departments in Germany in 2021^33^. As ED encounter episodes for the same reason of treatment can only be reimbursed once, we estimate the AKTIN infrastructure to encompass around 5% of all ED visits in the country. In some urban regions, the infrastructure has full coverage; one city has three competing public and private hospitals with EDs connected to the infrastructure within a 5-km radius.

All data access is currently free of charge for requesters and ED nodes. However, hospitals seeking active involvement in association bodies, such as the AKTIN DUAC, must become members of the AKTIN non-profit association, which currently incurs an annual fee of 250 €. The AKTIN infrastructure was established within a research project with a budget of €4.1 million. It is currently operated with annual infrastructure funding of €1.5 million provided by the Network University Medicine. 70 participating ED nodes received funding to establish the necessary CDA interfaces and currently receive annual infrastructure funding for their participation (not all of these ED nodes are currently connected to the AKTIN infrastructure and productive). However, 20 ED nodes participate at their own expense. The central components of the AKTIN Infrastructure are operated with a budget of about 500,000€ per year. A full-time software developer and a senior software engineer maintain the technical infrastructure and code base. IT project management and community management are overseen by two project coordinators, while two data scientists focus on data analysis and ensuring data quality. New services are financed through additional research projects and corresponding personnel.

## Discussion

Medical research networks avoid central data repositories; Storing data at the source is expected to reinforce data security and privacy compliance, thus empowering data holders. However, integrating data, as well as continuous and universal data querying is a governance challenge for the nodes, hindering broad secondary uses of EHRs^34^. In terms of our first objective, our results show that federated data access authorization allows the nodes of a nationwide research network to maintain local control over data, which in turn facilitates data access.

When scaling the AKTIN infrastructure, we found that having transparent operational frameworks allows the nodes to accommodate diverse local standards individually. This flexibility is critical during the local setup of components and when interacting with clinics and process owners, especially local data protection officers. Due to time constraints, data protection offices may delegate clearance of individual data queries to clinical end-users. Transparent governance mechanisms are then required to conduct spot checks. These results align with insights from the UK, highlighting the importance of robust data access pathways and standardized data access forms for population-wide research^8,20^.

A genuinely federated authorization process, with a power distribution between data providers, use-and-access processes, and data receivers, proved extremely helpful. In some regions, full coverage of ED nodes from competing private and public hospitals was achieved, underlining the level of trust in the framework. The ED registry and the ED nodes can authorize data access individually (Table 1). Our general strategy was to provide technical processes for maximum risk and allow the local nodes to adapt these within their local organizational processes. Including patient perspectives, however, was challenging due to anonymized data and the transient nature of emergency care. Instead, patient organizations are involved in research projects utilizing the AKTIN infrastructure.

**Table 1:** Responsibilities regarding data access authorization within the AKTIN infrastructure

	Central level responsibility	Local level responsibility
Safe projects	DUAC approves data access for specific research proposals.	Local ED nodes ensure that the research projects in which data are used align with local requirements.
Safe people	DUAC authorizes individuals and entities to access data.	Local ED nodes confirm the credentials and permissions of the data requester.
Safe data	DUAC ensures data access meets ethical and privacy standards. AKTIN IT implements queries according to the provisions of the DUAC.	Local ED nodes examine each query to validate its purpose and syntax. Clinical end-users provide context knowledge.
Safe settings	AKTIN TDAC guarantees data processing in secure environments.	The process owner and the hospital evaluate the operational framework within the AKTIN infrastructure.
Safe outputs	AKTIN TDAC oversees the release of aggregated or anonymized data. The DUAC reviews resulting publications.	Local ED nodes approve outputs for publication or further use.

The community-driven and interdisciplinary approach of the ED registry was the most effective strategy, facilitating widespread implementation and fast response rates within the technical system. Such an approach also enables the critical incorporation of clinical context knowledge, a prerequisite for any meaningful secondary analyses of EHR data. Data access authorization in clinical settings requires in-depth context knowledge from clinical end-users. The data access processes must not be overly time-consuming or complex. Structuring information to be easily comprehensible to clinical end-users was critical.

The increase in query volumes in the AKTIN infrastructure over the years demonstrates the successful adoption of the framework in various healthcare contexts. While the sample of connected EDs is not representative of the approximately 1500 EDs in Germany, the system still enables nationwide data access in the public and private sectors. Scalability is critical for sustaining the utility of medical research networks within a constantly changing healthcare landscape. We demonstrated that federated data access authorization can address communication barriers, limited access to information, and suboptimal data utilization in emergency medicine. These critical health system challenges are defined by the World Health Organization’s Classification of Digital Health Interventions^12^. Our implementation emphasizes the importance of robust authorization protocols within a secure trust architecture for overcoming the privacy-exploitation barrier. Our framework could, in principle, be used for data sharing across international borders, as it empowers the local nodes to authorize data access according to the local provisions. Such overarching standards are also present in international research networks operating across different national jurisdictions.

The accessed data is valuable for health services research^35^, prospective studies^36^, and artificial intelligence applications^37^, and was used to establish external quality management between ED through monthly benchmarking reports^38^. Further, a daily syndromic surveillance report has been introduced by the Robert Koch Institute, the German federal agency responsible for disease control and prevention^39^ The German Federal Ministry of Health utilizes data for weekly surveillance of ED capacities.

In terms of our second objective, our study shows that federated data access authorization can efficiently manage and handle data requests in research networks on a local level. While there is a high variability in the time until queries are addressed, most nodes answer queries relatively reliably within two weeks. We consider this response time within acceptable parameters, as each query requires an individual decision by the responsible stakeholders at each node. This process cannot be completely automated, as it requires explicit expert knowledge and a careful balancing of interests, which carries associated legal and ethical responsibilities.

Failed queries highlight persistent challenges in sharing healthcare data from EDs. These queries can primarily be attributed to syntax errors within the queries themselves or firewall issues. When data storage is federated, access to data is limited and based on assumptions. These may be invalid. The low rates of failed or retracted queries still indicate high system reliability, the appropriateness of the authorization process, and the clarity of the data request interface. Because anonymous data from system logs could only be used for evaluation, it was not possible to conduct a detailed analysis of query content, reasons for rejection, and usability of user interfaces. Further work is necessary to address the robustness of the technical process at the local and central nodes. A new data request management system is currently being developed^40^. The communication of HL7 FHIR resources will be supported in the future. However, CDA interfaces will be maintained to mitigate implementation costs for existing nodes.

Another limitation of our process is that it does not automatically ensure that the data content distributed to hospitals is secure, requiring manual checks. This dependence places significant trust in the integrity of data handlers, operating under the assumption that no malicious queries will be sent. Additionally, no standardized procedure for assessing and approving IT security measures exists. The IT security approvals in participating clinics are heterogeneous, leading to potential vulnerabilities where data handling protocols differ between nodes. Data breaches may also occur during the manual review of data queries when CSV files are generated, which can easily be shared or the system is incorrectly used. These shortcomings could be addressed on a technical level through privacy-preserving data processing, independent code reviews, or introducing a four-eyes principle.

Scaling data access in nationwide or international research networks requires transparent operational frameworks. Our approach shows that individuals and institutions gain more control over how their data is used by sharing data and joining federated systems. In this way, voluntary participation not only preserves autonomy but also expands the nodes’ capacity to shape outcomes. This aligns with the notion that freedom is not the absence of structure, but rather the ability to navigate and affect systems from within.

Our federated data access authorization framework is suitable for data sharing across different jurisdictions, potentially in other research networks worldwide. It enables secure, safe, and fast access to healthcare data from a wide range of healthcare contexts. While the sample of healthcare providers connected to the AKTIN infrastructure may not fully represent EDs in Germany, few systems in use offer continuous, universal, and nationwide access to EHRs. The system’s robustness, efficiency, and scalability make it a blueprint for future data-sharing initiatives in healthcare research. Integrating with initiatives like existing medical research networks is possible in principle but may require adaptation.

The system has particular practical relevance in the near future. Initiatives like the European Health Data Space aim to facilitate centralized access to health data across European countries. However, the question remains whether a learning healthcare system truly requires unrestricted data access, or if we should instead prioritize approaches like ours that emphasize fair data access by allowing data holders to maintain control over their data.

## Methods

For the first objective, we designed and implemented a technical system for federated data access authorization based on requirements gathered in 2016 in focus workshops with the initial 16 AKTIN project hospitals and a blueprint summary. For the second objective, we evaluated the efficiency of the system in a retrospective cohort study using KPIs obtained from log data during operation^41^.

### Blueprint summary

To achieve the first objective, we developed a service-oriented architecture, where centralized services offer specific functionalities (Supplementary Fig. 1). We implemented the architecture based on a broker design pattern and the Five Safes framework, which establishes five key components as a requirement for data access authorization—safe data, safe people, safe projects, safe settings, and safe outputs^42,43^.

A formal workflow description was developed and implemented within the technical components of the AKTIN infrastructure. We integrated the data access authorization workflow into the graphical user interface of the AKTIN DWH, the AKTIN DWH Manager. The AKTIN DWH backend is built as a Java EE web application, operating on WildFly with an embedded i2b2 instance. On the frontend side, the DWH can be administered through a web application—the DWH Manager*—*developed in AngularJS. All source code can be accessed on GitHub and is open source (https://www.github.com/aktin).

### System evaluation

Using KPIs, we retrospectively evaluated the efficiency and reliability of the data access authorization processes^41^ —objective two. Data for KPIs stemmed from anonymous user interaction data captured by the central AKTIN Broker as proprietary log files during live operation^44^. We extracted log files from the Broker and excluded *n* = 19 inactive or non-productive nodes (in total *n* = 77 nodes) from further analysis. Data included detailed timestamps for each state change of the data authorization workflow communicated with the AKTIN Broker between November 11, 2017, and October 21, 2024. Queries were typically communicated to a subset of relevant nodes; for example, pediatric emergency departments did not receive queries requesting data on adult patients.

To evaluate the systems in use, we differentiated between individual queries and repeated periodic queries (i.e., for daily syndromic surveillance of acute respiratory illness^39,45^ or monthly benchmarking reports^38^) with automatic rules for authorization. Queries contained technical (i.e., targeted source data validation^46^) and scientific requests (i.e., ^47^ or^35^). For the KPIs, we monitored the successful and failed queries as a general indicator of efficiency and reliability. Furthermore, we tracked the terminal state of queries, which includes the number of retractions or rejections, as well as withdrawn and unanswered queries, to provide a comprehensive view of the system’s usage. To address general efficiency, we evaluated the request volume per year and the time until the completion of queries.

We evaluated hospitals’ and data owners’ adoption and acceptance of the system on the basis of the number of systems in use and the number of EHRs integrated within the AKTIN DWHs—information we obtained from a voluntary survey for quality assurance practices and which 56 nodes participated.

The ethical committees of all participating institutions approved the operation of the infrastructure in accordance with the WMA Declaration of Helsinki (including the amendments made in Fortaleza and Taipei). The leading vote came from the Ethics Committee of Otto von Guericke University in Magdeburg with 160/15. No ethical review was required for the evaluation of KPIs, as neither personal data was processed nor any intervention was conducted.

## Supplementary information

Supplementary information

## Data Availability

Data used for the system evaluation are publicly available (10.5281/zenodo.14509530). The AKTIN DUAC terms of operation, the data protection concept, and a list of current research requests are available from www.aktin.org. The standardized emergency department medical record^17^ of the German Interdisciplinary Association for Intensive Care and Emergency Medicine is available from https://aktin.art-decor.pub/. The Robert Koch Institute, the German federal agency responsible for disease control and prevention publishes a daily syndromic surveillance report available from https://public.data.rki.de/t/public/views/Notaufnahmesurveillance/DashboardSyndrome^48^ and the German Federal Ministry of Health publishes Data for weekly surveillance of ED capacities available from https://infektionsradar.gesund.bund.de/de/gesamt/notaufnahmen.

## Code availability

All source code of the AKTIN infrastructure can be accessed on GitHub and is open source (https://www.github.com/aktin). Source code for the system evaluation and exemplary SQL requests are publicly available from GitHub (https://github.com/aktin/publications/tree/main/jbienzeisler/federated_authorization). Analyses were performed using R version 4.3.1.
