# Supplementary material for: Implementation report on pioneering federated data access for the German National Emergency Department Data Registry
Source: NPJ Digit Med. 2025 Feb 11;8:94. doi: 10.1038/s41746-025-01481-w (PMC11814142; doi:10.1038/s41746-025-01481-w)
Supplement: Supplementary file 1 — Supplementary information [file 41746_2025_1481_MOESM1_ESM.pdf]

Supplementary Information

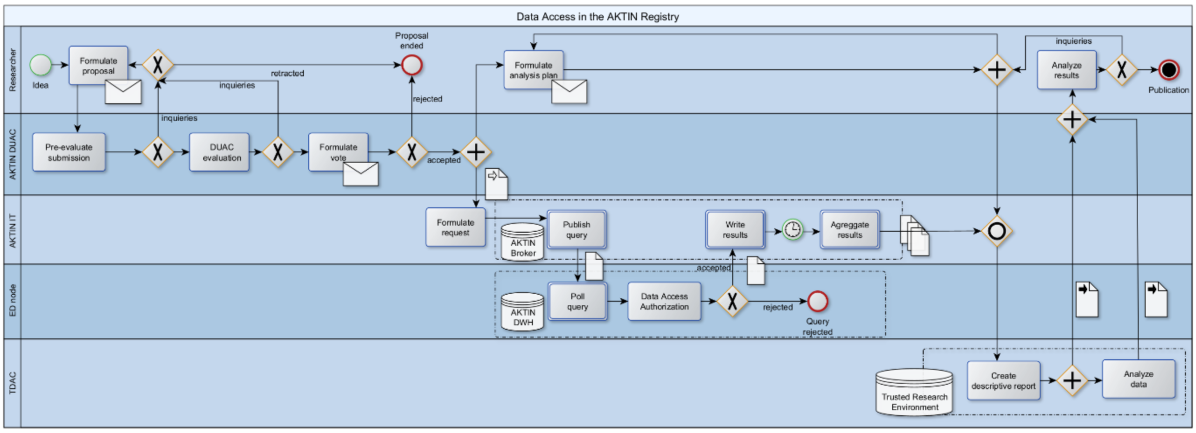

Supplementary Figure 1: Data Access in the AKTIN Emergency Department Data Registry: Data can be requested according to an established data access protocol upon approval from the AKTIN Data Use and Access Committee (DUAC). Using the AKTIN Broker, datasets are queried from local nodes—the AKTIN Data Warehouse—where each query must be individually reviewed. Data can be analyzed in the trusted research environment maintained by the AKTIN Trusted Data Analysis Center. Note: This diagram employs a modified version of BPMN, adapted to clarify specific workflow elements unique to the AKTIN infrastructure. This adaptation may not strictly adhere to formal BPMN guidelines.

Supplementary Table 1: System Requirements

ID	Requirement Description	User Story	Stakeholder	Notes
1	Data sovereignty and local control	As a data owner, I want to maintain control over data access to ensure compliance with local regulations and individual decision-making processes.	Hospital	Reduces the risk of strategic vulnerability to competitors and data breaches for hospitals.
2	Federated data access authorization	As a data owner, I want a qualified authorization and evaluation of each data access request.	Hospital	Balances risks (data breaches) with benefits (improved data governance, transparency, research).
3	Varying stakeholder roles in data access	As a process owner, I want to have a transparent overview of who authorized data requests to ensure accountability.	Process Owner	Supports streamlined data governance and transparency, addressing liability concerns.
4	Secure and transparent operational framework	As a process owner, I need a secure system that ensures data integrity, data provenance, and procedural visibility to comply with regulations.	Process Owner	Essential for data integrity, reducing privacy breaches and misuse of data.
5	Qualified review of local data and queries	As a process owner, I want to be able to review and validate data queries to ensure they align with local standards before sharing results.	Process Owner	Reduces potential liability for data breaches.
6	Simplified authorization process for end-users	As a user, I want a straightforward interface to authorize data access requests quickly to manage my workload effectively.	User	Mitigates increased workload for users.
7	Optional automatic rules for recurring authorizations	As a user, I want to set up rules to automate repetitive data access authorizations to reduce manual effort.	User	Mitigates increased workload for users.
8	Email notifications for authorization events	As a user, I want to receive notifications when data access requests require my action to stay informed without constantly checking the system.	User	Ensures that users can focus on critical tasks, reducing administrative burden.
9	Data export in accessible formats	As a user, I need the ability to export query results in formats like CSV for further analysis and reporting.	User	Allow users to evaluate data queries
10	Workflow state transitions for data requests	As a user, I want to see the state of data requests at each step to monitor progress and address any issues that arise.	User	Improves accountability for process owners and mitigates the risk of data mismanagement.

11	Error handling in data authorization	As a user, I want clear feedback in case of errors during the data authorization process to quickly identify and resolve issues.	User	Reduces errors in data handling, ensuring a more reliable process for all stakeholders.
12	Continuous access to data stored in AKTIN DWHs for predefined purposes	To operate the AKTIN infrastructure, we need continuous access to data stored in AKTIN DWHs	AKTIN Infrastructure	Essential for maintaining the functionality and objectives of the AKTIN infrastructure.
13	Five Safes	To operate the AKTIN infrastructure, we need to provide the participating nodes with the necessary information to atuhorize data access	AKTIN Infrastructure	Provide information according to the five safes framework
14	Asynchronous data transmission across nodes	To operate the AKTIN infrastructure, we need the system to support asynchronous data transmission to handle data integration from multiple sources seamlessly.	AKTIN Infrastructure	Enables access to continuous data from different hospitals.
15	Support for multiple data formats	To operate the AKTIN infrastructure, we need the system to accommodate various data formats to ensure compatibility with the locally used and i2b2-based AKTIN DWH.	AKTIN Infrastructure	Enhances the adaptability of data integration across nodes.
16	Long-term data usability with open-source solutions	To operate the AKTIN infrastructure, we need to use open-source solutions to ensure the sustainability and longevity of the data management system.	AKTIN Infrastructure	Allows for sustainable operation and long-term data usability

Supplementary Table 2: Query Volume per Year and number of ED cases integrated within the AKTIN infrastructure.

Year	N ED Nodes	N Total ED Cases	N Requests	N Queries	N Individual Queries	N Periodic Queries
2017*	12	425,229	2	15	15	0
2018	16	593,097	25	225	209	16
2019	17	825,815	60	529	111	418
2020	21	754,537	424	5,719	217	5,502
2021	46	1,018,809	668	10,827	463	10,364
2022	50	1,523,266	586	14,630	862	13,768
2023	54	1,570,503	629	20,982	1,045	19,937
2024*	58	1,271,382	566	23,340	1,131	22,209
**Total**	**58**	**7,982,638**	**2,960**	**76,267**	**4,054**	**72,214**

**Data captured for 2017 and 2024 did not cover the entire year.*

Supplementary Table 3: Analysis of Key Performance Indicators derived from log files of the AKTIN Broker. Individual queries and periodically repeated queries are differentiated. For individual queries, data access has to be authorized individually. For periodic queries, emergency departments may omit future authorization and grant automatic data access authorization for future and past queries.

	**Individual Query (N=4,053)**	**Periodic Query (N=72,214)**	**Total (N=76,267)**
**Last communicated status**	..	..	..
completed	3,234 (79.8%)	62,888 (87.1%)	66,122 (86.7%)
failed	114 (2.8%)	722 (1.0%)	836 (1.1%)
interaction	3 (0.1%)	16 (0.0%)	19 (0.0%)
processing	2 (0.0%)	97 (0.1%)	99 (0.1%)
queued	5 (0.1%)	102 (0.1%)	107 (0.1%)
rejected	121 (3.0%)	732 (1.0%)	853 (1.1%)
retrieved	574 (14.2%)	7,657 (10.6%)	8,231 (10.8%)
**Days until completed**	..	..	..
Mean (SD)	16.5 (38.2)	8.6 (23.5)	9.0 (24.5)
Median (Range)	3.8 (0.0, 510.2)	3.6 (0.0, 481.0)	3.6 (0.0, 510.2)
Q1, Q3	0.3, 15.4	0.7, 6.6	0.7, 7.3
IQR	15.1	5.9	6.6
**Days until rejected**	..	..	..
Mean (SD)	74.1 (88.7)	21.4 (53.2)	23.6 (56.1)
Median (Range)	38.1 (0.0, 367.8)	-0.0 (-0.0, 584.7)	-0.0 (-0.0, 584.7)
Q1, Q3	12.2, 105.0	-0.0, 0.1	-0.0, 12.0
IQR	92.8	0.1	12.0

Supplementary Table 4: Checklist of iCHECK-DH guidelines

Item	Section	Description	Line in Manuscript
1	TITLE	Identification as an implementation report, and description of the implementation in the title and/or keywords	Title
2	ABSTRACT	Provide a summary of key elements, including implementation strategy, intervention, and KPIs/Outputs	29-38
3	INTRODUCTION	Context: Describe geographical areas, organizations, target populations, and implementation context	61-67
4		Problem statement: Description of the healthcare or public health problem addressed by the implementation	107-119
5		Similar Interventions: Mention inspiration and added value compared to other implementations	120-130
6	METHODS	Aims and Objectives: Describe main objectives, outcomes, and KPIs	336-339
7		Blueprint summary: Design, key features of the intervention, and implementation strategy	341-347
8		Technical Design: Reasons for tool development, description of functionality, technology type, and integration	345-350
9		Target: Characteristics of the targeted group, site, or system	352-355
10		Data: Data governance, data protection measures, patient consent, and data hosting	353-358
11		Interoperability: Interfaces, standards used, and rationale for choice	353-356
12		Participating entities: Description of organizations, partners, funders, and ownership	336-339

13		Budget Planning: Planned budget, costs, funding, and budget duration	236-238
14		Sustainability: Business model, sustainability model, and exit strategies	236-247
15	RESULTS	Coverage: Implementation coverage (international, national, regional) and relative importance	231-235
16		Outcomes: Primary and other outcome(s) of the implementation	193-226
17		Lessons learned: Success factors, challenges, budget adherence, and recommendations	249-276
18		Unintended consequences: Description of any unintended consequences, harms, or negative side-effects	227-230
19	DISCUSSION	Conclusion: Summary of the conclusions and future implications	277-232
20	GENERAL	General: Statement(s) on regulatory approvals, ethical considerations, and conflicts of interest	372-404

Supplementary Note 1: SQL Query Sent to ED Node in xml data structure (as visualized in Figure 3). The query syntax is visible within the query view when clicking "See Syntax".

```
<sql xmlns="http://aktin.org/ns/i2b2/sql">

    <temporary-table name="temp_encounter_data"/>

    <temporary-table name="temp_diagnoses"/>

    <temporary-table name="temp_cohort_data"/>

    <temporary-table name="temp_sample"/>



    <source type="application/sql">

        <![CDATA[

        -- Preprocessing of raw data

        CREATE TEMPORARY TABLE temp_cohort_data AS



        -- SELECT BASIC INFORMATION

        SELECT DISTINCT

            obs.encounter_num AS encounter_num,

            obs.patient_num AS patient_number,

            EXTRACT(year FROM age(vis_dim.start_date, pat_dim.birth_date)) AS
age_in_years,

            pat_dim.sex_cd as gender,

            vis_dim.start_date AS admission_date,

            vis_dim.end_date AS discharge_date

        FROM i2b2crcdata.observation_fact obs

        JOIN i2b2crcdata.patient_dimension pat_dim ON obs.patient_num =
pat_dim.patient_num

        JOIN i2b2crcdata.visit_dimension vis_dim ON obs.encounter_num =
vis_dim.encounter_num



        -- Column Time Filter
```

```
        WHERE vis_dim.start_date BETWEEN '20230101' AND '20240101';

        -- End Column Time Filter



        CREATE TEMPORARY TABLE temp_sample AS

        SELECT (SELECT COUNT(DISTINCT encounter_num) FROM temp_cohort_data) AS
total_aktin_cases;



        -- Filter for VALID Cases

        -- get patient ID for patient number

        ALTER TABLE temp_cohort_data ADD COLUMN patient_identifier VARCHAR;

        UPDATE temp_cohort_data SET patient_identifier = pat.patient_ide

        FROM i2b2crcdata.patient_mapping pat

        WHERE temp_cohort_data.patient_number = pat.patient_num;



        -- get encounter ID for encounter number

        ALTER TABLE temp_cohort_data ADD COLUMN encounter_identifier VARCHAR;

        UPDATE temp_cohort_data SET encounter_identifier = enc.encounter_ide

        FROM i2b2crcdata.encounter_mapping enc

        WHERE temp_cohort_data.encounter_num = enc.encounter_num;



        -- get billing ID for encounter

        ALTER TABLE temp_cohort_data ADD COLUMN billing_identifier VARCHAR;

        UPDATE temp_cohort_data SET billing_identifier = obs.tval_char

        FROM i2b2crcdata.observation_fact obs

        WHERE temp_cohort_data.encounter_num = obs.encounter_num

        AND concept_cd LIKE 'AKTIN:Case%';



        -- Drop all AKTIN opt-outs from temp_cohort_data
```

```
        -- get AKTIN patient ID

        ALTER TABLE temp_cohort_data ADD COLUMN patient_opt VARCHAR;

        UPDATE temp_cohort_data SET patient_opt = opt.pat_psn

        FROM i2b2crcdata.optinout_patients opt

        WHERE ((opt.study_id = 'AKTIN' AND opt.optinout = 'O') OR (opt.study_id
= 'CERT' AND opt.optinout = 'I'))

        AND opt.pat_ref = 'PAT'

        AND opt.pat_psn = temp_cohort_data.patient_identifier;



        -- get AKTIN encounter ID

        ALTER TABLE temp_cohort_data ADD COLUMN encounter_opt VARCHAR;

        UPDATE temp_cohort_data SET encounter_opt = opt.pat_psn

        FROM i2b2crcdata.optinout_patients opt

        WHERE ((opt.study_id = 'AKTIN' AND opt.optinout = 'O') OR (opt.study_id
= 'CERT' AND opt.optinout = 'I'))

        AND opt.pat_ref = 'ENC'

        AND opt.pat_psn = temp_cohort_data.encounter_identifier;



        -- get AKTIN billing ID

        ALTER TABLE temp_cohort_data ADD COLUMN billing_opt VARCHAR;

        UPDATE temp_cohort_data SET billing_opt = opt.pat_psn

        FROM i2b2crcdata.optinout_patients opt

        WHERE ((opt.study_id = 'AKTIN' AND opt.optinout = 'O') OR (opt.study_id
= 'CERT' AND opt.optinout = 'I'))

        AND opt.pat_ref = 'BIL'

        AND opt.pat_psn = temp_cohort_data.billing_identifier;



        -- Delete rows merged with opt-in/out patients

        DELETE FROM temp_cohort_data
```

```
        WHERE patient_opt IS NOT NULL

        OR encounter_opt IS NOT NULL

        OR billing_opt IS NOT NULL;



        -- Delete columns

        ALTER TABLE temp_cohort_data DROP COLUMN patient_opt, DROP COLUMN en-
counter_opt, DROP COLUMN billing_opt, DROP COLUMN billing_identifier, DROP COL-
UMN patient_identifier, DROP COLUMN encounter_identifier;



        -- Add a column to store the count of valid cases

        ALTER TABLE temp_sample ADD COLUMN valid_aktin_cases INTEGER;

        -- Update the filtered_cases column with the count of distinct encounter
numbers from temp_cohort_data

        UPDATE temp_sample SET valid_aktin_cases = (SELECT COUNT(DISTINCT en-
counter_num) FROM temp_cohort_data);



        -- ### Table 1: Encounter Data (without diagnoses) ###

        CREATE TEMPORARY TABLE temp_encounter_data AS

        SELECT DISTINCT



        -- Column Age_Years

        age_in_years,

        -- End Column Age_Years

        -- Column Gender

        gender as gender,

        -- End Column Gender

        -- Column Admission Date

        TO_CHAR(admission_date, 'YYYY-MM-DD HH24') AS admission_timestamp,

        -- End Column Admission Date
```

```
        -- Column Transfer/Discharge

        AGE(discharge_date, admission_date) AS discharge_time,



        encounter_num

        FROM temp_cohort_data

        -- Sort table by encounter number in ascending order

        ORDER BY encounter_num ASC;



        -- Column Assignment

        ALTER TABLE temp_encounter_data ADD COLUMN referral_type VARCHAR;

        UPDATE temp_encounter_data SET referral_type = CASE

            WHEN substr(concept_cd, 16) = 'VAP' THEN 'Contracted Doctor/Prac-
tice'

            WHEN substr(concept_cd, 16) = 'KVNPIK' THEN 'Emergency Practice at
Hospital'

            WHEN substr(concept_cd, 16) = 'KVNDAK' THEN 'Emergency Service Out-
side of Hospital'

            WHEN substr(concept_cd, 16) = 'RD' THEN 'Rescue Service'

            WHEN substr(concept_cd, 16) = 'NA' THEN 'Emergency Doctor'

            WHEN substr(concept_cd, 16) = 'KLINV' THEN 'Clinic/Transfer'

            WHEN substr(concept_cd, 16) = 'NPHYS' THEN 'Non-physician Referral'

            WHEN substr(concept_cd, 16) = 'OTH' THEN 'Other'

            ELSE

                substr(concept_cd, 16)

            END

        FROM i2b2crcdata.observation_fact

        WHERE concept_cd LIKE 'AKTIN:REFERRAL%' AND modifier_cd = '@'

        AND i2b2crcdata.observation_fact.encounter_num = temp_encounter_data.en-
counter_num;
```

```
        -- End Column Assignment


        -- Column Transport

        ALTER TABLE temp_encounter_data ADD COLUMN transport_mode VARCHAR;

        UPDATE temp_encounter_data SET transport_mode = CASE

            WHEN concept_cd = 'AKTIN:TRANSPORT:1' THEN 'Ambulance'

            WHEN concept_cd = 'AKTIN:TRANSPORT:2' THEN 'Emergency Ambulance'

            WHEN concept_cd = 'AKTIN:TRANSPORT:3' THEN 'Emergency Doctor Vehi-
cle'

            WHEN concept_cd = 'AKTIN:TRANSPORT:4' THEN 'Rescue Helicopter'

            WHEN concept_cd = 'AKTIN:TRANSPORT:NA' THEN 'None'

            WHEN concept_cd = 'AKTIN:TRANSPORT:OTH' THEN 'Other'

            ELSE

                concept_cd

            END

        FROM i2b2crcdata.observation_fact

        WHERE concept_cd LIKE 'AKTIN:TRANSPORT%' AND modifier_cd = '@'

        AND i2b2crcdata.observation_fact.encounter_num = temp_encounter_data.en-
counter_num;

        -- End Column Transport


        -- Column Presentation Reason (cedis)

        ALTER TABLE temp_encounter_data ADD COLUMN presentation_reason VARCHAR;

        UPDATE temp_encounter_data SET presentation_reason = CASE

            WHEN concept_cd = '75322-8:UNK' THEN '999'

            WHEN concept_cd = 'CEDIS30:UNK' THEN '999'

            WHEN concept_cd LIKE '75322-8%' THEN substr(concept_cd, 8)

            WHEN concept_cd LIKE 'CEDIS%' THEN substr(concept_cd, 9)
```

```
            ELSE

                concept_cd

            END

        FROM i2b2crcdata.observation_fact

        WHERE (concept_cd LIKE 'CEDIS%' OR concept_cd LIKE '75322-8%') AND modi-
fier_cd = '@'

        AND i2b2crcdata.observation_fact.encounter_num = temp_encounter_data.en-
counter_num;

        -- End Column Presentation Reason



        -- Column Initial Triage

        ALTER TABLE temp_encounter_data ADD COLUMN initial_triage VARCHAR;

        UPDATE temp_encounter_data SET initial_triage = CASE

            WHEN concept_cd LIKE 'MTS:%' THEN substr(concept_cd, 5)

            WHEN concept_cd LIKE 'ESI:%' THEN substr(concept_cd, 5)

            WHEN concept_cd LIKE 'AKTIN:ASSESSMENT%' THEN substr(concept_cd, 18)

            ELSE

                concept_cd

            END

        FROM i2b2crcdata.observation_fact

        WHERE (concept_cd LIKE 'MTS%' OR concept_cd LIKE 'ESI%' OR concept_cd
LIKE 'AKTIN:ASSESSMENT%') AND modifier_cd = '@'

        AND i2b2crcdata.observation_fact.encounter_num = temp_encounter_data.en-
counter_num;

        -- End Column Initial Triage



        -- Column Triage System

        ALTER TABLE temp_encounter_data ADD COLUMN triage_system VARCHAR;

        UPDATE temp_encounter_data SET triage_system = CASE
```

```
            WHEN concept_cd LIKE 'MTS:%' THEN 'MTS'

            WHEN concept_cd LIKE 'ESI:%' THEN 'ESI'

            WHEN concept_cd LIKE 'AKTIN:ASSESSMENT' THEN 'Other'

            ELSE

                concept_cd

            END

        FROM i2b2crcdata.observation_fact

        WHERE (concept_cd LIKE 'MTS%' OR concept_cd LIKE 'ESI%' OR concept_cd
LIKE 'AKTIN:ASSESSMENT%') AND modifier_cd = '@'

        AND i2b2crcdata.observation_fact.encounter_num = temp_encounter_data.en-
counter_num;

        -- End Column Triage System



        -- Column Disposition

        ALTER TABLE temp_encounter_data ADD COLUMN disposition VARCHAR;

        UPDATE temp_encounter_data SET disposition = CASE

            WHEN concept_cd = 'AKTIN:TRANSFER:1' THEN 'Admission to Functional
Area'

            WHEN concept_cd = 'AKTIN:TRANSFER:2' THEN 'External Transfer to
Functional Area'

            WHEN concept_cd = 'AKTIN:TRANSFER:3' THEN 'Admission to Monitoring
Station'

            WHEN concept_cd = 'AKTIN:TRANSFER:4' THEN 'External Transfer to Mon-
itoring Station'

            WHEN concept_cd = 'AKTIN:TRANSFER:5' THEN 'Admission to Normal Ward'

            WHEN concept_cd = 'AKTIN:TRANSFER:6' THEN 'External Transfer to Nor-
mal Ward'

            WHEN concept_cd = 'AKTIN:DISCHARGE:1' THEN 'Death'

            WHEN concept_cd = 'AKTIN:DISCHARGE:2' THEN 'Discharge Against Medi-
cal Advice'

            WHEN concept_cd = 'AKTIN:DISCHARGE:3' THEN 'Treatment Discontinued
by Patient'
```

```
            WHEN concept_cd = 'AKTIN:DISCHARGE:4' THEN 'Discharge to Home'

            WHEN concept_cd = 'AKTIN:DISCHARGE:5' THEN 'Discharge to Continuing
Care Physician'

            WHEN concept_cd = 'AKTIN:DISCHARGE:6' THEN 'No Doctor Contact'

            WHEN concept_cd = 'AKTIN:DISCHARGE:OTH' THEN 'Other Type of Dis-
charge'

            ELSE

                concept_cd

            END

        FROM i2b2crcdata.observation_fact

        WHERE ((concept_cd LIKE '%TRANSFER%' AND concept_cd <> 'AKTIN:TRANS-
FER:Time of Transfer') OR concept_cd LIKE '%DISCHARGE%') AND modifier_cd = '@'

        AND i2b2crcdata.observation_fact.encounter_num = temp_encounter_data.en-
counter_num;

        -- End Column Disposition



        -- Column Triage Timestamp

        ALTER TABLE temp_encounter_data ADD COLUMN triage_timestamp TIMESTAMP;

        UPDATE temp_encounter_data SET triage_timestamp = start_date

        FROM i2b2crcdata.observation_fact

        WHERE (concept_cd LIKE 'MTS%' OR concept_cd LIKE 'ESI%' OR concept_cd
LIKE 'AKTIN:ASSESSMENT') AND modifier_cd = 'effectiveTimeLow'

        AND i2b2crcdata.observation_fact.encounter_num = temp_encounter_data.en-
counter_num;

        -- End Column Triage Timestamp



        -- Column First Doctor Contact Timestamp

        ALTER TABLE temp_encounter_data ADD COLUMN first_doctor_con-
tact_timestamp TIMESTAMP;

        UPDATE temp_encounter_data SET first_doctor_contact_timestamp =
start_date

        FROM i2b2crcdata.observation_fact
```

```
        WHERE (concept_cd = 'AKTIN:PHYSENCOUNTER' OR concept_cd = 'AKTIN:First
Doctor Contact Time') AND modifier_cd = 'timeLow'

        AND i2b2crcdata.observation_fact.encounter_num = temp_encounter_data.en-
counter_num;

        -- End Column First Doctor Contact Timestamp



        -- Column AKTIN Diagnose Table

        -- ### Table 2: Patient Diagnoses (Emergency Department Dataset) ###

        CREATE TEMPORARY TABLE temp_diagnoses AS

        SELECT DISTINCT

            temp_cohort_data.encounter_num,

            instance_num,

            substr(i2b2crcdata.observation_fact.concept_cd, 9) as icd_code

        FROM temp_cohort_data

        INNER JOIN i2b2crcdata.observation_fact

        ON temp_cohort_data.encounter_num = i2b2crcdata.observation_fact.encoun-
ter_num

        WHERE i2b2crcdata.observation_fact.concept_cd LIKE 'ICD10GM:%' AND
i2b2crcdata.observation_fact.provider_id = '@' and i2b2crcdata.observa-
tion_fact.modifier_cd = '@';



        -- Column primary emergency room diagnosis

        ALTER TABLE temp_diagnoses ADD COLUMN leading_diagnosis VARCHAR;

        UPDATE temp_diagnoses SET leading_diagnosis = 'f'

        FROM i2b2crcdata.observation_fact

        WHERE concept_cd LIKE 'ICD10GM:%' AND provider_id = '@' AND modifier_cd
= 'AKTIN:DIAG:F'

        AND i2b2crcdata.observation_fact.encounter_num = temp_diagnoses.encoun-
ter_num

        AND i2b2crcdata.observation_fact.instance_num = temp_diagnoses.in-
stance_num
```

```
        AND substr(i2b2crcdata.observation_fact.concept_cd, 9) = temp_diagno-
ses.icd_code;

        -- End Column primary emergency room diagnosis



        -- Column diagnosis markers

        ALTER TABLE temp_diagnoses ADD COLUMN additional_diagnosis_marker VAR-
CHAR;

        UPDATE temp_diagnoses SET additional_diagnosis_marker = substr(modi-
fier_cd, 12)

        FROM i2b2crcdata.observation_fact

        WHERE concept_cd LIKE 'ICD10GM:%'

        AND provider_id = '@'

        AND (modifier_cd LIKE 'AKTIN:DIAG:%' AND modifier_cd NOT LIKE 'AK-
TIN:DIAG:F')

        AND i2b2crcdata.observation_fact.encounter_num = temp_diagnoses.encoun-
ter_num

        AND i2b2crcdata.observation_fact.instance_num = temp_diagnoses.in-
stance_num

        AND substr(i2b2crcdata.observation_fact.concept_cd, 9) = temp_diagno-
ses.icd_code;

        -- End Column diagnosis markers



        -- Delete empty cases and instance numbers

        ALTER TABLE temp_diagnoses DROP COLUMN instance_num;

        DELETE FROM temp_diagnoses WHERE icd_code is NULL;

        -- End Column AKTIN Diagnose Table

        ]]>

    </source>

    <anonymize>

        <key table="temp_encounter_data" column="encounter_num"/>

        <ref table="temp_diagnoses" column="encounter_num"/>
```

```
    </anonymize>

    <export table="temp_encounter_data" destination="case_data"/>

    <export table="temp_diagnoses" destination="diag_data"/>

    <export table="temp_sample" destination="sample"/>

</sql>
```
